# Structural Insights Into the Nuclear Import of Gallid Alphaherpesvirus 1 Large Tegument Protein

**DOI:** 10.1002/mbo3.70216

**Published:** 2026-01-22

**Authors:** Babu Kanti Nath, Crystall M. D. Swarbrick, Reuben Blades, Daryl Ariawan, Ole Tietz, Gualtiero Alvisi, Jade K. Forwood, Subir Sarker

**Affiliations:** ^1^ Biosecurity Research Program and Training Centre, Gulbali Institute Charles Sturt University Wagga Wagga New South Wales Australia; ^2^ Dementia Research Centre, Macquarie Medical School, Faculty of Medicine, Health and Human Sciences Macquarie University Sydney New South Wales Australia; ^3^ Department of Molecular Medicine University of Padua Padua Italy; ^4^ Training Hub Promoting Regional Industry and Innovation in Virology and Epidemiology, Gulbali Institute Charles Sturt University Wagga Wagga New South Wales Australia; ^5^ Biomedical Sciences & Molecular Biology, College of Medicine and Dentistry James Cook University Townsville Queensland Australia; ^6^ Australian Institute of Tropical Health and Medicine James Cook University Townsville Queensland Australia; ^7^ Department of Microbiology, Anatomy, Physiology and Pharmacology, School of Agriculture, Biomedicine and Environment La Trobe University Melbourne Victoria Australia

**Keywords:** crystallography, Gallid alphaherpesvirus 1, importins, nuclear trafficking

## Abstract

Gallid alphaherpesvirus 1 (GaAHV‐1), also referred to as infectious laryngotracheitis virus (ILTV), primarily targets the upper respiratory tract of chickens. This infection leads to significant economic setbacks worldwide in the poultry sector, driven by reductions in egg output, weight gain, and increased mortality rates. Even with the broad implementation of vaccination programs, ILTV outbreaks remain a challenge, as vaccine strains can revert to a virulent form under field conditions. This underscores the need to explore targeted therapeutic options, including a deeper understanding of GaAHV‐1's nuclear trafficking mechanisms, critical for viral replication. The herpesvirus large tegument protein UL36 contains N‐terminal nuclear localization signals (NLSs) that are essential for capsid routing to the nuclear pore complex (NPC). However, the mechanisms by which UL36 of GaAHV‐1 mediates nuclear import remain poorly understood. In this study, we identified the NLS of GaAHV‐1 UL36 and elucidated their binding mechanism with human nuclear import proteins. Using high‐resolution crystal structures and quantitative assays, we mapped the specific residues and regions within UL36's N‐terminal domain that facilitate binding to importin (IMP) α. Moreover, we revealed variations in binding affinities among different importin isoforms. Our biochemical and structural analyses demonstrate that the predicted N‐terminal NLS of GaAHV‐1 UL36 is critical for IMPα binding. These findings provide detailed molecular insights into the interaction between the GaAHV‐1 large tegument protein and IMPs, paving the way for the development of targeted antiviral therapies.

## Graphical abstract

The study identifies and characterizes a bipartite nuclear localization signal within the GaAHV‐1 UL36 large tegument protein, revealing its structural interaction with importin *α*/*β*1 and confirming a conserved mechanism of nuclear import through biochemical, crystallographic, and mutational analyses.

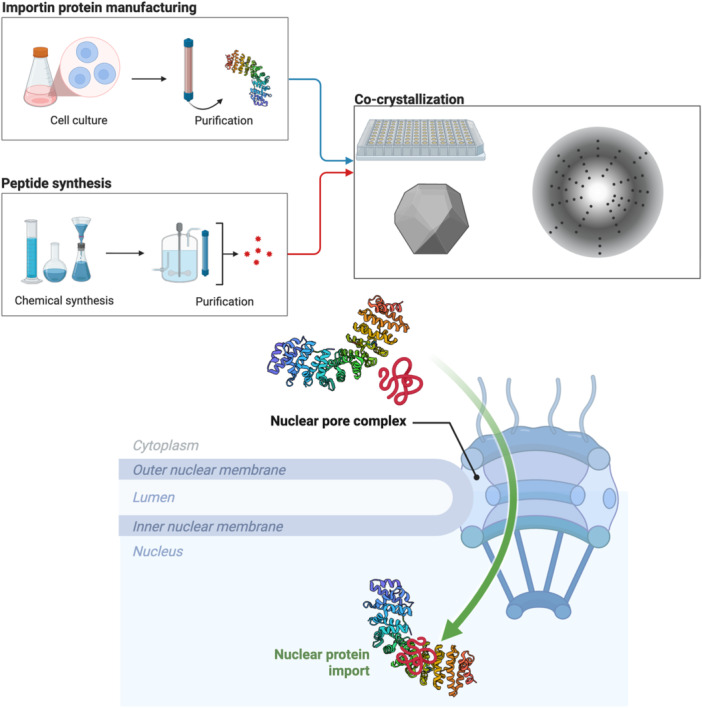

## 1 Introduction

Infectious laryngotracheitis (ILT) is a highly transmissible respiratory illness that affects chickens, pheasants, and peafowl, and is caused by Gallid alphaherpesvirus 1 (GaAHV‐1) (Guy and Garcia 2009). Previously termed ILT virus (ILTV), GaAHV‐1 is classified under the genus *Iltovirus*, within the subfamily *Alphaherpesvirinae* of the family *Herpesviridae* (Lee et al. 2010). The virus is predominantly excreted via respiratory secretions and can spread efficiently through aerosol inhalation or indirect contact via contaminated equipment and personnel. Clinically, ILT presents with signs such as conjunctivitis, nasal discharge, and a drop in egg production, while more severe infections may include hemorrhagic tracheitis, gasping, coughing, and the expulsion of blood‐stained mucus. The disease has a worldwide distribution, with morbidity and mortality rates largely influenced by the virulence of the infecting strain (Kirkpatrick et al. 2006; Oldoni et al. 2009). Additionally, factors include the virus load present in the environment and concurrent infections with other respiratory pathogens (Guy and Garcia 2009). Mortality rates can reach as high as 70%, leading to significant economic losses, particularly in regions with high‐density poultry production (Bagust et al. 2000). Presently, there is no specific antiviral treatment for ILT, and disease management relies mainly on vaccination. Available vaccines—such as live attenuated strains (Alls et al. 1969; Thilakarathne et al. 2019), recombinant viral vector‐based vaccines (Davison et al. 2006; Johnson et al. 2010) and recombinant deletion mutant formulations (Thilakarathne et al. 2019; Devlin et al. 2007) offer varying levels of protection. However, residual virulence in some vaccine strains and recombination events between vaccine and wild‐type viruses remain significant concerns. Therefore, advancing our understanding of GaAHV‐1 nuclear trafficking mechanisms is vital for the development of more effective therapeutic strategies.

The transport of protein cargo into the nucleus is facilitated by a specialized group of nuclear transport receptors known as importins (IMPs), along with auxiliary proteins such as Ran GTPase and nucleoporins (Macara 2001; Bednenko et al. 2003; Stewart 2007). IMPβ1 or one of its several orthologues recognize cargoes and import them into the nucleus. Several different IMPβl recognize different NLSs, and that the best characterized‐pathway is dependent on the IMPβ1/IMPα heterodimer whereby IMPα recognizes cargos directly and IMPβ1 mediates translocation across the NPC (Cingolani et al. 2002; Wing et al. 2022; Fontes et al. 2000). Based on sequence homology, IMPα proteins are divided into three subfamilies. In humans, these subfamilies include: α1 (comprising IMPα5/KPNA1, α6/KPNA5, and α7/KPNA6), α2 (including IMPα1/KPNA2 and α8/KPNA7), and α3 (containing IMPα3/KPNA4 and α4/KPNA3) (Miyamoto et al. 2016); Pumroy and Cingolani 2015). The core region of IMPα features 10 Arm repeats, hydrophobic motifs approximately 42–43 amino acids in length that form the binding interface for cNLS‐bearing cargos. These signals interact with two key regions within the Arm domain: the major NLS binding site (Arm repeats 2–4) and the minor binding site (Arm repeats 6–8). The main contact points for binding are designated P1–P5 (major site) and P1′–P4′ (minor site). Monopartite cNLSs, such as the one from the SV40 large T antigen, typically bind only the major site, while bipartite cNLSs, such as that of nucleoplasmin, engage both sites (Stewart 2007). The IMPα/β1‐mediated nuclear import pathway is the most extensively studied mechanism for nuclear entry of proteins. After cargo recognition by one of the seven known IMPα isoforms, the cargo‐IMPα complex associates with IMPβ1 via the N‐terminal region of IMPα. The resulting trimeric complex is guided through the NPC by interactions between IMPβ1 and phenylalanine‐glycine (FG)‐rich motifs present on nucleoporins (Cingolani et al. 1999; Milles et al. 2015). Once inside the nucleus, RanGTP facilitates the dissociation of the complex, enabling cargo release and recycling of the IMPs back to the cytoplasm for subsequent rounds of Vogel et al. 2024; Bischoff and Görlich 1997; Lee et al. 2005).

Herpesvirus capsids must traverse the cytoplasm and dock at the NPCs, where capsid structural rearrangements, through mechanisms yet to be fully understood, facilitate genome release and transport into the nucleus (Döhner et al. 2023). This process which is best characterized for HSV‐1 allows the transcription of the immediate early genes (Flint et al. 2020). A key player in this phase is the large tegument protein VP1‐2, encoded by the UL36 gene, which is both essential and highly conserved throughout the *Herpesviridae* family (Desai 2000; Fuchs et al. 2004; Lee et al. 2006; Luxton et al. 2006). VP1‐2 is a multifunctional protein involved in several pivotal stages of the viral life cycle, including viral entry, intracellular capsid transport, and virion assembly (Desai 2000; Shanda and Wilson 2008; Abaitua et al. 2012). In HSV‐1, a functional NLS has been identified near the N‐terminal ubiquitin‐specific protease (USP) domain of VP1‐2 (residues 400–420: GLPKRRRPTWTPPSSVEDLTS) (Abaitua et al. 2012; Kosugi et al. 2009a; Hennig et al. 2014). Since deletion of such NLS prevented capsid docking at the NPCs and start of viral gene expression (Abaitua et al. 2012), but did not affect the assembly and release of extracellular virions, it has been suggested that VP1‐2 nuclear targeting is essential during the early phase of the virus life cycle (Abaitua et al. 2012). Because such NLS is positionally conserved across several herpesvirus orthologues (Kosugi et al. 2009a), it is believed to play a similar crucial role in the early infection stages of all herpesviruses (Hennig et al. 2014).

Despite its apparent significance, the specific IMPs that interact with this NLS have not been characterized, and the functional relevance of analogous NLSs in other *Alphaherpesvirinae*, especially in viruses infecting animal species remains poorly understood. This study aims to characterize the structural and functional attributes of the predicted NLS within the VP1‐2 protein of GaAHV‐1 using a combination of structural and biochemical approaches.

## 2 Methods

### 2.1 Large Tegument Gene Sequence Comparison and Phylogenetic Analysis

Amino acid sequences of UL36 orthologues available in GenBank were retrieved and analyzed using Base‐by‐Base (Hillary et al. 2011) and Geneious Prime (version 2023.1.1). Sequence similarity was assessed through Base‐by‐Base and the MAFFT software package (version 11.0.11) (Version 11.0.11) (Hillary et al. 2011; Katoh and Standley 2013). Multiple sequence alignments were performed using MAFFT (version 7.450), applying the G‐INS‐i algorithm with a gap opening penalty of 1.53 and an offset value of 0.123. To infer evolutionary relationships, a maximum‐likelihood (ML) phylogenetic tree was generated in Geneious using the general time‐reversible (GTR) model and 1000 bootstrap replicates to assess branch support.

### 2.2 Peptide and Gene Construct Design and Synthesis

**Figure 1:**
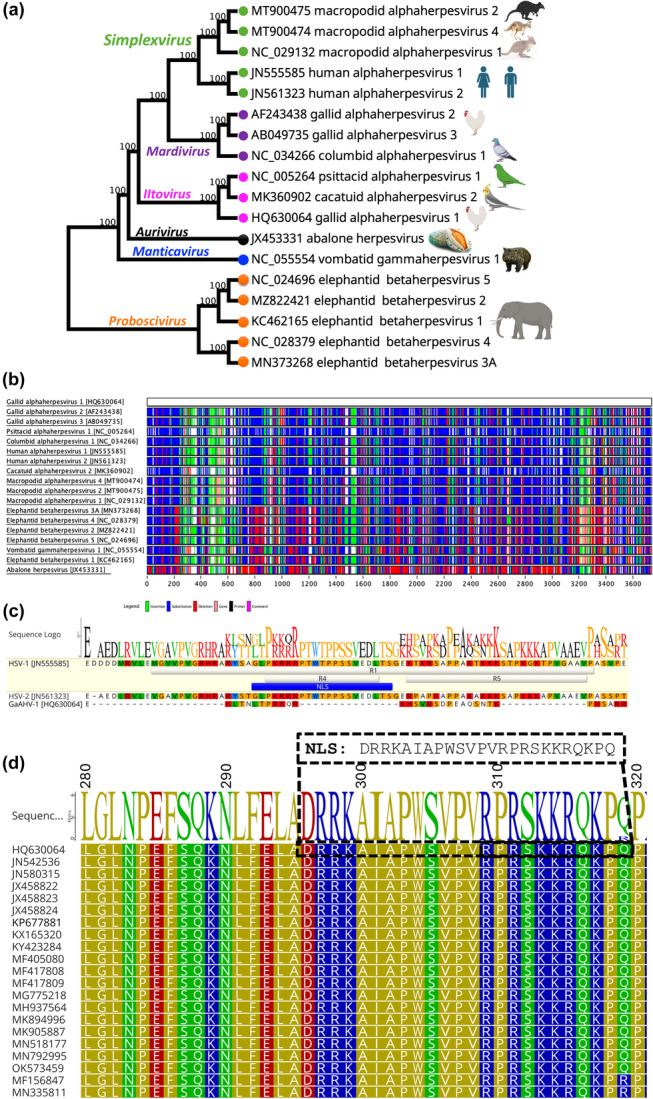
Evolutionary comparison and genetic variability of the selected UL36 gene. (a) Phylogenetic relationships between GaAHV‐1 and others selected herpesviruses. Maximum likelihood (ML) phylogenetic tree was generated using multiple sequence alignments of the UL36 gene within Geneious Prime (version 2023.1.1). Bootstrap support values are shown as percentages next to the corresponding branches. Each tip on the tree is labeled with the GenBank accession number followed by the associated virus name. (b) A comparative visualization of UL36 gene amino acid sequences from selected herpesviruses was carried out using Base‐By‐Base. Sequence variations within the UL36 gene of GaAHV‐1 are color‐coded: green indicates insertions, blue denotes substitutions, red represents deletions, black identifies primers, and pink highlights user comments (please refer to the zoomed‐in view in Supporting Informtormation S1: Figure S6) (c) Comparison of R1 region encoding NLS of HSV‐1 (residues 375–455) and ‐2 (Hennig et al. 2014) with corresponding region of GaAHV‐1. (d) Simplified diagram displaying the conservation of the identified putative NLS regions of GaAHV‐1 UL36.

UL36 sequences of the selected GaAHV‐1 were downloaded from GenBank and potential NLS were predicted by using cNLS mapper program (Kosugi et al. 2009b). Our analysis predicted a putative bipartite NLS with a score of > 5.0 (^296^DRRKAIAPWSVPVRPRSKKRQKPQ^319^) (Figure 1). A synthetic peptide corresponding to the predicted amino acid sequence, modified at the N‐terminus with FITC/Ahx, was synthesized at Macquarie University (Sydney, Australia) using standard Fmoc‐based solid‐phase peptide synthesis protocols on a CEM Liberty Blue Peptide Synthesizer (CEM, USA). Initially, rink amide resin was pre‐swelled for 1 h in a 1:1 mixture of dimethylformamide (DMF) and dichloromethane (DCM). Amino acids were prepared at a concentration of 0.2 M in DMF before being introduced into the synthesis workflow. Peptide chains were assembled from the C‐terminus to the N‐terminus using a 3‐min coupling cycle at 90°C, with five equivalents of each amino acid, 10 equivalents of the activator (0.5 M DIC in DMF), and five equivalents of the activator base (a solution of 0.5 M Oxyma and 0.05 M DIPEA in DMF). Fmoc deprotection was carried out using 20% piperidine in DMF for 2 min at 90°C, followed by resin washes with DMF. Arginine residues were double‐coupled to ensure complete incorporation. Following final Fmoc deprotection of N‐terminal amino hexanoic acid (Ahx), resin was removed from synthesizer, transferred to a syringe fitted with a propylene filter, washed, and FITC coupling performed. FITC is coupled using three equivalents of FITC and six equivalents of DIPEA in DMF overnight. Peptides were washed with DMF, DCM, and methanol before cleavage. Cleavage from the resin was performed using a cocktail composed of 92.5% trifluoroacetic acid (TFA), 2.5% triisopropylsilane (TIPS), 2.5% thioanisole, and 2.5% water for 3–6 h at room temperature. The cleaved peptides were precipitated in ice‐cold diethyl ether, dissolved in water, freeze‐dried, and purified by reverse‐phase high‐performance liquid chromatography (HPLC) on a Shimadzu LC‐20AD system (Shimadzu, Japan). Mass spectrometry analysis was performed using a Shimadzu LCMS‐8050 in positive electrospray ionization mode, fitted with a Polaris 3 C18‐A column (150 × 4.6 mm, Agilent Technologies, USA) (Supporting Information S1: Figure S1). Mutants of this FITC‐tagged NLS peptides were engineered based on structural data of IMPα interaction sites to selectively disrupt binding at the major site, minor site, or both. Additionally, truncated forms of IMPα isoforms lacking the N‐terminal importin β‐binding (IBB) domain were used in this study, including hIMPα1ΔIBB (His‐tagged, TEV‐cleavable), mIMPα2ΔIBB (His‐tagged, TEV‐cleavable), and hIMPα3ΔIBB (His‐tagged, TEV‐cleavable), along with IMPβ1 encoded in the pET30a vector—all previously described in the literature (Munasinghe et al. 2022; Teh et al. 1999). A list of peptides use is shown in Supporting Information S1: Table S2 and Supporting Information S1: Figures S1–S5.

### 2.3 Recombinant Expression and Purification

Human IMPα1ΔIBB (UniProt: P52292), mouse IMPα2ΔIBB (UniProt: P52293), human IMPα3ΔIBB (UniProt: O00629), and mouse IMPβ1 (UniProt: P70168) were overexpressed in *E. coli* pLysS cells using an auto‐induction protocol (Studier 2005). After a 36‐h growth at room temperature, the cells were collected by centrifugation at 5232 × g and resuspended in His buffer A (50 mM phosphate buffer, 300 mM NaCl, 20 mM imidazole, pH 8) at 15 mL per 2 L of culture. Cells were lysed through two freeze‐thaw cycles. The resulting lysate was treated with 1 mL of 20 mg/mL lysozyme (Sigma‐Aldrich, USA) and 10 µL of 50 mg/mL DNase (Sigma‐Aldrich, USA) per 50 mL of suspension, then incubated at room temperature for 1 h on a tube roller. Soluble proteins were separated by centrifugation at 11,269 × g for 30 min and the supernatant was filtered through a 0.45 µm low‐protein‐binding filter. The filtrate was loaded onto a 5 mL HisTrap HP column (GE Healthcare, USA) and washed with 20 column volumes of His buffer A using an AKTApurifier FPLC system (GE Healthcare, USA). Proteins were eluted with a linear gradient of imidazole ranging from 20 to 500 mM (ChemSupply, Australia), and eluted fractions were pooled. Further purification was achieved via size‐exclusion chromatography using a HiLoad 26/60 Superdex 200 column (GE Healthcare, USA) pre‐equilibrated with GST buffer A (50 mM Tris, 125 mM NaCl). Fractions corresponding to the expected molecular weights were collected, concentrated using an Amicon Ultra centrifugal filter unit with a 10 kDa cutoff (Merck Millipore, USA), aliquoted, and stored at −80°C. The purity of the protein preparations was evaluated by SDS‐PAGE, running samples on a 4%–12% Bis‐Tris Plus gel (Thermo Fisher Scientific) at 165 V for 30 min before use in downstream applications.

### 2.4 Crystallization, Data Collection and Structure Determination

mIMPα2ΔIBB was crystallized by the hanging drop vapor diffusion method in 300 μL reservoir of 0.6 M sodium citrate, 0.1 M HEPES pH 7.0 and 10 mM DTT at 23°C (Jagga et al. 2021). Individual rod‐shaped crystals were formed within 2 days of incubation. These crystals were soaked with the peptide of interest and cryoprotected using the reservoir solution supplemented with 20% glycerol before being flash cooled in liquid nitrogen. X‐ray diffraction data were collected at the Australian Synchrotron using the MX2 beamline and an Eiger 16 M detector (Aragão et al. 2018). Data processing, including indexing and integration, was carried out with MOSFLM (Battye et al. 2011), and further merging, scaling, and R_free calculations were conducted using AIMLESS from the CCP4 suite (Evans 2011). Refinement and model building were performed iteratively using COOT (Emsley et al. 2010) and Phenix (Adams et al. 2010). The crystal structure was solved by molecular replacement using Phaser (McCoy et al. 2007) with PDB entry 1IQ1 as the search template for IMPα. The final model was validated and deposited in the Protein Data Bank (PDB ID: 9MIK), with additional details provided in Supporting Information S1: Tables S2 and S3.

### 2.5 Fluorescence Polarization (FP) Assays

FP assays were performed according to the protocol described previously (Moerke 2009; Nematollahzadeh et al. 2024). In brief, FITC‐conjugated peptides (2 nM) were incubated with serially diluted concentrations of IMPα (starting at 20 μM) across 23‐wells, each well containing 200 μL of GST Buffer A (50 mM Tris, 125 mM NaCl). FP readings were taken using a CLARIOstar Plus plate reader (BMG Labtech, Germany). Each experiment was independently repeated three times, and a no‐importin control was included to verify specificity. The average data from all three replicates was used to generate binding curves using GraphPad Prism (version 9.3.1).

### 2.6 Electro‐Mobility Shift Assay (EMSAs)

EMSAs were conducted using a previously described protocol (Nematollahzadeh et al. 2024; Nath et al. 2025). FITC‐labeled peptides (10 μM) were incubated with 20 μM of each IMPα isoform in a 20 μL reaction volume, which included 3 μL of 50% glycerol and was completed with GST Buffer A (50 mM Tris, 125 mM NaCl). Samples were electrophoresed on 1.5% agarose gels prepared in TB buffer (45 mM Tris base, 45 mM boric acid, pH ~8.5) at 70 V for 90 min. Fluorescence images were captured using the SYBR green filter of a Bio‐Rad Gel Doc imaging system (Bio‐Rad Laboratories, USA). Gels were subsequently stained with Coomassie Brilliant Blue R‐250 staining solution (40% ethanol, 10% acetic acid, 0.2% dye) for 10 min and destained overnight in a solution of 10% ethanol and 10% acetic acid before final imaging.

## 3 Results

### 3.1 Genetic Variability and Phylogenetic Relationship of the GaAHV‐1 UL36 Gene

The largest tegument protein of herpesvirus, pUL36, serves as a critical multivalent cross‐linker, connecting viral capsids to both the tegument and associated membrane proteins during viral assembly. Upon entry into host cells, pUL36 also facilitates the release of incoming capsids from the outer tegument and the viral envelope (Schipke et al. 2012). Despite its essential roles, the mechanisms underlying the nuclear trafficking of pUL36 remain poorly understood. To investigate this, we analyzed representative herpesvirus genomes and extracted full‐length UL36 sequences for comparative analysis and further characterization.

The amino acid sequence of the UL36 protein from GaAHV‐1 was found to be highly divergent compared to those of other selected herpesviruses (Figure 1, Supporting Information S1: Table S1). Among the analyzed species, GaAHV‐1 UL36 showed the highest pairwise sequence identity with cacatuid alphaherpesvirus 2 (34.37%), followed by psittacid alphaherpesvirus 1 (33.83%). Phylogenetic analysis based on the UL36 gene (Figure 1a) strongly supports the closest evolutionary relationship between GaAHV‐1 and other avian herpesviruses, including cacatuid alphaherpesvirus 2 and psittacid alphaherpesvirus 1, within the genus *Iltovirus*. However, the protein sequence length of pUL36 varies significantly between HSV‐1 (3139 residues, GenBank accession no. JN555585) and GaAHV‐1 (2784 residues, GenBank accession no. HQ630064), with only approximately 20.4% sequence identity (Figure 1B).

Moreover, GaAHV‐1 UL36 lacks the corresponding NLS of HSV‐1 (_400_GLPKRRRPTWTPPS SVEDLTS_420_) (Figure 1C). To further investigate sequence conservation, we analyzed 21 full‐length UL36 genes from GaAHV‐1 and found them to be highly similar, with amino acid sequence identities of 99% to 100%. To better understand the nuclear transport mechanisms of GaAHV‐1 UL36, we performed a bioinformatic analysis using cNLS Mapper and manual inspection to identify putative NLSs. This analysis revealed a putative NLS within a highly conserved region (positions 296–319 in the alignment), which showed nearly 100% amino acid identity across all 21 UL36 genes analyzed (Figure 1d). Notably, the predicted NLS in GaAHV‐1 was not present in HSV‐1 (GenBank accession no. JN555585). In contrast, GaAHV‐1 lacks any considerable conservations with the previously characterized NLS of UL36 of HSV‐1 (_400_GLPKRRRPTWTPPSSVEDLTS_420_) (Luxton et al. 2006; Shanda and Wilson 2008; Abaitua et al. 2012).

### 3.2 Biochemical Determination of GaAHV‐1 NLS Preference for Both Human IMPα and IMPβ1 Isoforms

**Figure 2:**
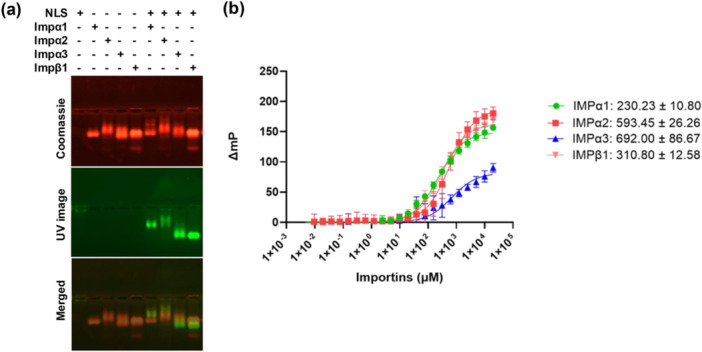
GaAHV‐1 large tegument protein NLS binds to several IMPs (a) EMSA showing binding of GaAHV‐1 large tegument protein NLS to IMPα isoforms and IMPβ1. GaAHV‐1 large tegument protein NLS peptides contain a FITC and Ahx linker and were visualized by excitation with an UV lamp (green). Protein bands were visualized post‐electrophoresis using Coomassie Blue staining (red). The EMSA results reflect outcomes from three independent replicates. (b) FP assay quantifying the binding affinity of the GaAHV‐1 NLS to different IMP isoforms. Results represent the mean ± standard error (SE) from three separate experiments. Dissociation constants (Kd values) were determined by conducting a saturation binding experiment using FITC‐labeled peptide and importin proteins. The resulting data are then fitted to a binding curve using non‐linear regression analysis in GraphPad Prism.

To evaluate whether the predicted GaAHV‐1 bipartite NLS bind to host nuclear import receptors IMPα and IMPβ1, and to identify any preference for specific receptor isoforms, we conducted biochemical binding assays. EMSAs were performed to qualitatively assess the interactions between the GaAHV‐1 NLS and various importin isoforms, including IMPα family members (*α*1, *α*2, *α*3) and IMPβ1. Three independent experiments confirmed that the predicted NLS was capable of binding to IMPα isoforms as well as to IMPβ1. It is evident that there is minimal shifting of IMPs upon binding to the NLS, which is not unexpected in the Coomassie‐stained gels, as importin is a large protein of approximately 52 kDa. However, the shift of the FITC‐labeled NLS after binding to importin is clearly visible in the UV image (Figure 2a). To further assess the interactions and determine the binding affinities of the IMP/peptide complexes, quantitative FP assays were performed following established methods (Nematollahzadeh et al. 2024; Alvisi et al. 2023; Athukorala et al. 2024; Cross et al. 2023; Cross et al. 2023; Hoad et al. 2023) (Figure 2b). The bipartite NLS demonstrated higher binding affinities, interacting with IMPα1 (*K_D_* = 230 nM) and IMPβ1 (*K_D_* = 311 nM), followed by IMPα2 (*K_D_* = 593 nM) and IMPα3 (*K_D_* = 692 nM). These findings indicate that GaAHV‐1 NLS can bind to different IMPs with varying affinities and supports the notion that GaAHV‐1 UL36 can bind to either the IMPα adapter, or directly to the IMPβ1 nuclear import receptors, to mediate nuclear import.

### 3.3 The High‐Resolution Crystal Structure Reveals the Binding Interface of Mouse IMPα2 and GaAHV‐1 NLS

**Figure 3:**
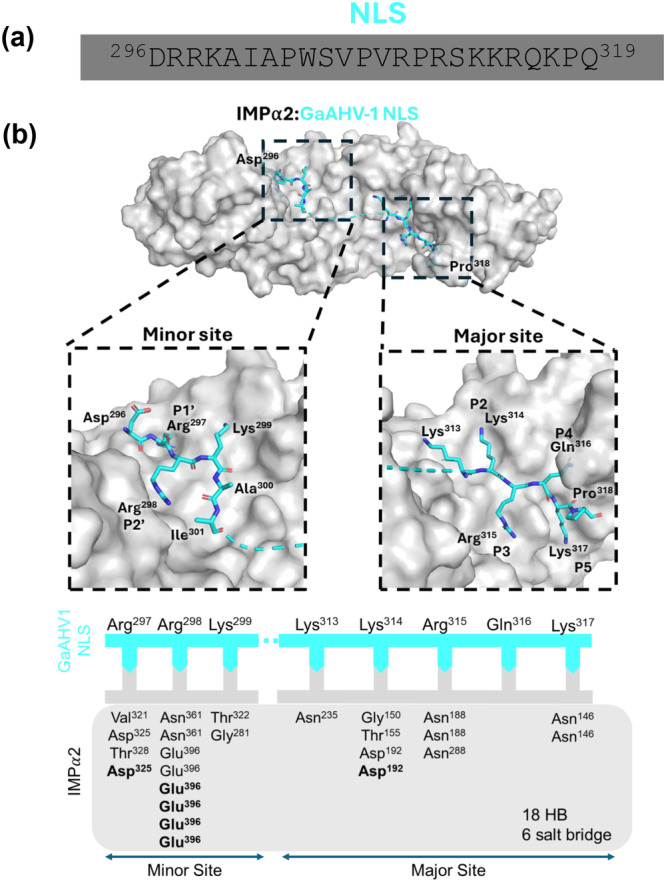
Crystal structure and binding interactions of GaAHV‐1 NLS in complex with IMPα2. (a) Sequence of the predicted NLS of GaAHV‐1. (b) Top panel: Schematic overview of the GaAHV‐1 protein and structure of GaAHV‐1 NLS (cyan sticks) and IMPα2 (gray surface) complex resolved to 2.6 Å resolution. The zoomed‐in images illustrate critical residues of GaAHV‐1 NLS binding in both minor and major IMPα2 sites. This structure has been deposited in the PDB and given the code: 9MIK. Bottom panel: Simplified representation of IMPα and GaAHV‐1 NLS binding interactions. The GaAHV‐1 NLS (cyan line) residues bound to IMPα2 (gray box) are indicated through complementary arrows. Residues in bold denote salt bridge and non‐bold residues indicate hydrogen bonds identified using the PDBePISA server.

To explore the molecular mechanisms by which GaAHV‐1 enters the nucleus, we co‐crystallized predicted bipartite nuclear localization signal (GaAHV‐1 NLS; residues 296–319) in complex with the nuclear import receptor IMPα2. Crystallization was achieved using the hanging‐drop vapor diffusion technique, yielding well‐formed rod‐shaped crystals in the presence of the NLS peptide. Diffraction data were collected at the MX2 beamline of the Australian Synchrotron, and the structure was resolved at a resolution of 2.8 Å. The data were indexed in space group *P2₁2₁2₁*, with unit cell dimensions of 78, 90, and 100 Å for *a*, *b*, and *c*, respectively, and angles of 90° for *α*, *β*, and *γ*. Structure determination was carried out via molecular replacement using Phaser (McCoy et al. 2007) by molecular replacement with PDB 6BW1 as the search model. The IMPα2:NLS structure contained one molecule of IMPα2 and two chains of GaAHV‐1 NLS. Iterative rounds of refinement and modeling were performed with Phenix (Adams et al. 2010) and COOT (Emsley et al. 2010; Adams et al. 2010), with a final *R*_work_/*R*_free_ of 0.17/0.21. The full data collection and refinement statistics are given in Supplementary Table S2. The final IMPα2: NLS model contained IMPα2 (residues 72–498), with GaAHV‐1 NLS residues ^313^KKRQKP^318^ bound at the major site (ARM 2–4) and GaAHV‐1 NLS residues ^297^RRK^299^ bound the minor site of IMPα2. Structural characterization of the interface revealed that GaAHV‐1 tegument protein NLS binds to IMPα as a canonical bipartite NLS, containing a lysine at the major site P2 position (Figure 3b, Supporting Information S1: Table S3). The IMPα2:NLS structure reveals the minor site to be occupied by an “RR” motif similar to the Hendra and Nipah virus W NLS:IMPα2 structures (Smith et al. 2018) rather than the canonical ‘KR’ motif typically seen with bipartite NLS, such as AAV (Hoad et al. 2024) and MERS ORF4b (Munasinghe et al. 2022). Within the minor site GaAHV‐1 NLS Arg^297^ hydrogen bonds with IMPα2 Val^321^, Asp^325^, and Thr^328^, and forms a salt bridge with IMPα2 Asp^325^ in the P1′ site (Figure 3b; Supporting Information S1: Table S3 and S4). GaAHV‐1 NLS Arg^298^ occupies the P2′ site and hydrogen bonds with IMPα2 Asn^361^ and Glu^396^, as well as forming salt bridges with IMPα Glu^396^. Within the P3′ site the GaAHV‐1 NLS Lys^299^ hydrogen bonds with IMPα2 Thr^322^ and Gly^281^. The flexibility of the linker region spanning residues 302–311 likely prevented it from being resolved in the structure, due to its inherently dynamic nature. The major site was clearly resolved with GaAHV‐1 NLS residues occupying the P1–P5 sites of IMPα2. The canonical P2 site was occupied with the GaAHV‐1 NLS Lys^314^ hydrogen bonding to IMPα2 Gly^150^, Thr^155^ and Asp^192^ and forming a salt bridge with Asp^192^. The P3 site is occupied by GaAHV‐1 NLS Arg^315^ interacting with IMPα through hydrogen bonds with Asn^188^ and Asn^288^. The P5 site is occupied by the GaAHV‐1 NLS Lys^317^ which holds hydrogen bonds with IMPα2 Asn^146^.

### 3.4 Mutational Studies Confirm Bipartite Nature of GaAHV‐1 NLS

**Figure 4:**
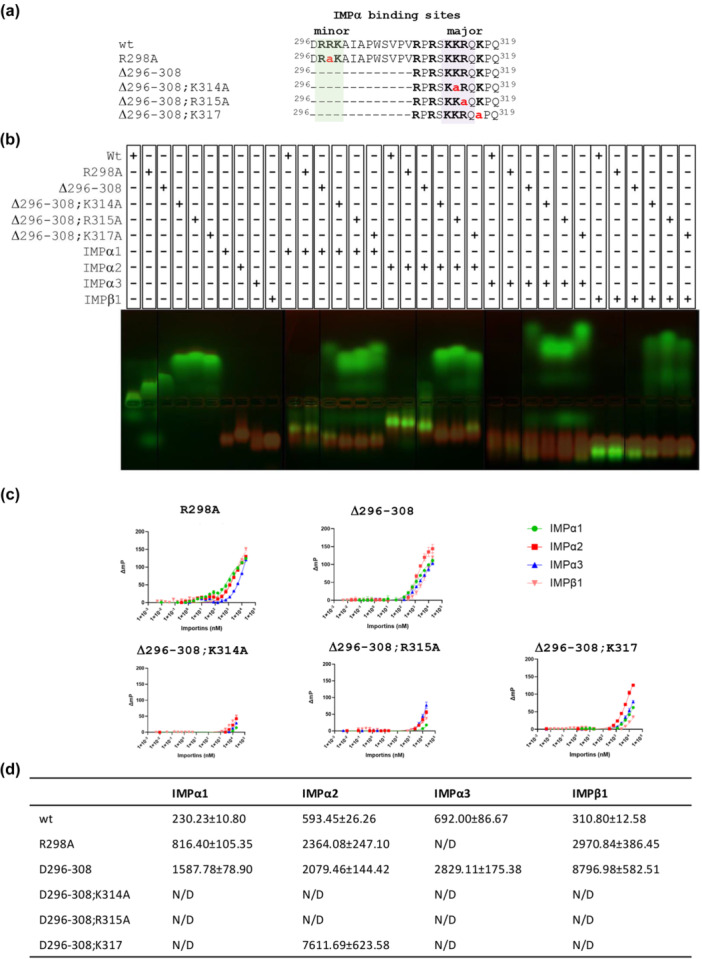
Binding affinities of importin isoforms with GaAHV‐1 tegument protein NLS mutants. (A) Schematic representation of GaAHV‐1 tegument protein NLS mutants. (B) EMSA showing poor binding affinity of GaAHV‐1 NLS mutant peptides to IMPα isoforms and IMPβ1. The peptides contain a FITC and Ahx linker and were visualized by excitation with an UV lamp (green). Protein bands were visualized post‐electrophoresis using Coomassie Blue staining (red). The figure supplied here is the overlay image of UV and Coomassie stained image. The EMSA results reflect outcomes from three independent replicates. (C) FP assay measuring the direct binding between the NLS mutants and the indicated IMPs isoforms. (D) Kd values are shown are mean + standard error of the mean (SE) relative to three independent experiments. The Kd value was determined by conducting a saturation binding experiment using FITC‐labeled peptide and importin proteins. The resulting data were then fitted to a binding curve using non‐linear regression analysis in GraphPad Prism.

Since GaAHV‐1 residues ^296^DRRKAIAPWSVPVRPRSKKRQKPQ^319^ comprise two basic stretches of amino acids (^297^ **RRK** ^299^) and (^314^ **KR**Q**K** ^317^), which could be visualized interacting with IMPα2 minor and major binding sites, respectively (Figure 4A), we hypothesized that they could represent a bipartite NLS. In order to investigate this possibility, we assessed the role of the minor‐site interacting residues on binding of GaAHV‐1 NLS to several IMPs (Figure 4A), by both EMSA (Figure 4B) and FP (Figure 4C). The R298A substitution, targeting a key interaction in the P2′ pocket in IMPα minor binding site, reduced co‐migration of the GaAHV‐1 NLS;R298A peptide with all IMPα isoforms tested in EMSA (Figure 4B and Supporting Information S1: Figure S7), and significantly decreased the Kd of interaction (Figure 4C,D), confirming the importance of minor site interaction for GaAHV‐1 NLS interaction with IMPα. A complete deletion of the upstream basic stretch of amino acids further decreased, but not completely abolished binding of the NLS peptide to all IMPs tested, as exemplified by the residual shift observed in EMSA upon incubation of GaAHV‐1 NLS;Δ296‐308 peptide with IMPs (Figure 4B), and the further increased in Kd measured by FP (Figure 4C,D). These results suggest that GaAHV‐1 NLS interaction with IMPα minor binding site as mediated by residues 297–299 is important for optimal interaction with IMPs. Intriguingly when key residues at the major binding site were also substituted to Ala, interaction with IMPs was almost completely abolished, as shown by failure of peptides GaAHV‐1 NLS;Δ296‐308;K314A, GaAHV‐1 NLS;Δ296‐308;R315A and GaAHV‐1 NLS;Δ296‐308;K317A to co‐migrate with IMPs in EMSAs (Figure 4B) and efficiently bind IMPs in FP assays (Figure 4C,D). Altogether, our results reveal the importance of upstream and downstream GaAHV‐1 NLS basic residues for interaction with several IMPs, strongly suggesting its bipartite nature.

## 4 Discussion

Herpesviruses are DNA viruses that replicate within the cell nucleus of host cells, a process that requires understanding viral protein behavior and localization to develop effective antiviral strategies (Weller and Coen 2012; Packard and Dembowski 2021). Herpesviruses are complex viruses, with multiple proteins that enter the nucleus to facilitate replication (Copeland et al. 2009; Döhner et al. 2021; Alvisi et al. 2013). Studies on HSV‐1 have identified that it expresses 21 proteins predominantly located in the cytoplasm or associated with cytoplasmic membranes, while 16 proteins exhibit nuclear or subnuclear localization. Additionally, several other viral proteins are distributed across both the nucleus and cytoplasm (Xing et al. 2011). Notably, envelope proteins from herpesviruses are generally localize in the cytoplasm, whereas capsid proteins tend to accumulate within the nucleus. This pattern suggests that the intracellular positioning of each protein likely reflects its role in the viral life cycle, particularly during replication (Xing et al. 2011).

HSV‐1 utilizes multiple nuclear localization mechanisms during infection (Ojala et al. 2000; Goldfarb et al. 2004; Görlich and Kutay 1999). For example, HSV‐1 relies on the IMPα/β1 pathway to ensure the nuclear localization of its DNA polymerase processivity factor UL42 (Alvisi et al. 2008), whereas the tegument viral protein VP16 of HSV‐1 interacts with the host cell factor HCF‐1 for nuclear localization (Boissiere 1999). However, the exact mechanism of nuclear transport in GaAHV‐1 has remained unclear. To explore this, we analyzed the classical NLS region in the large tegument protein of GaAHV‐1, employing structural and biophysical approaches to understand its interactions with cellular IMPs.

Previous work with HSV‐1 indicates that the VP1‐2 NLS is essential for infection via capsid routing to the NPC (Abaitua et al. 2012). However, the biochemical and structural characterization of alphaherpesvirus UL36 NLS interactions with IMPs remains unexplored. In this study, we characterized the UL36‐IMPα2 complex and identified a unique bipartite NLS in GaAHV‐1. An analysis of the GaAHV‐1 large tegument protein revealed one predicted bipartite NLS with an overlapping central region. Biochemical analysis demonstrated binding of FITC‐labeled NLS peptide with all tested IMPα isoforms and IMPβ1. The predicted NLS interacted with IMPα1 and IMPβ1 with higher affinity, followed by IMPα2 and IMPα3. These findings suggest that GaAHV‐1 large tegument protein can bind to different IMPs through NLS within this basic cluster region, supporting its ability to enter the nucleus via multiple nuclear import pathways. In the conventional pathway, proteins containing an NLS are recognized by IMPα, which then associates with IMPβ1 to mediate translocation through the nuclear pore complex. In contrast, unconventional pathways bypass the NLS and IMPα/β1 machinery, utilizing alternative mechanisms such as passive diffusion, direct binding to nucleoporin protein at the NPC, and IMPβ1 dependent pathways for nuclear entry (Wagstaff and Jans 2009; Poon and Jans 2005). To further investigate these possibilities, the high‐resolution crystal structures of the binding interface between IMPα2 and GaAHV‐1 NLS were resolved. GaAHV‐1 NLS is a bipartite NLS with an RR motif in the minor site and canonical lysine (K^314^) in the P2 position of the major site.

Among alphaherpesviruses, the N‐terminal basic cluster forms a continuous sequence, allowing it to function as a monopartite motif in some species (such as HSV, PRV, and equine herpesvirus 1 [EHV‐1]). Interestingly, all alphaherpesvirus VP1‐2 NLS motifs retain a long linker structure and a defined P/S/T‐rich element, which may contribute to their function under certain conditions (Hennig et al. 2014). Bipartite NLS motifs typically interact with IMPα which has two binding sites for NLS motifs (a major and a minor binding pocket) (Chang et al. 2013). The linker region between the upstream and downstream clusters can make contacts with IMPα, potentially influencing binding. Since neurons may express different nuclear import machinery, the unique organization of NLS in alphaherpesviruses might not be essential for replication in culture, but it could be important for transport, import, and replication in vivo (Hennig et al. 2014). Intriguingly a similar bipartite architecture is conserved in HSV‐1 DNA polymerase catalytic subunit UL30 and its processivity factor UL42, whereby bipartite NLSs were described, in stark contrasts with the monopartite NLSs characterized in *β*‐ and *γ*‐ herpesviruses (Alvisi et al. 2013, 2008, 2005, 2006, 2007).

In the EMSA analysis assessing mutations within the GaAHV‐1 NLS region predicted to interact with the major binding sites of IMPα, the mutant peptides exhibited only weak interactions with IMPα isoforms (Figure 4b). This suggests that these specific residues contribute to active NLS in a cellular context. Supporting this, FP assays demonstrated that substitutions at key residues such asLys^314^A, Arg^315^A, and Lys^317^A, significantly diminished binding across all tested IMPα isoforms (Figure 4c–d). Together, these findings support a model in which residues within the major binding site of the NLS are critical for interaction with IMPs, highlighting a binding preference among IMPα isoforms. This observation aligns with previous findings where detergent‐treated HSV‐1 virions could bind nuclear pore complexes (NPCs) in vitro (Ojala et al. 2000), and such binding was partially inhibited by antibodies targeting either nucleoporins or IMPβ1, suggesting a role for IMPβ1 in facilitating capsid association with the nuclear envelope, although no specific viral receptor was identified (Ojala et al. 2000). Despite this, the specificity of NLS interactions with importin isoforms remains poorly defined. Given that structural analyses reveal highly conserved NLS binding grooves among IMPα isoforms, and that relative NLS binding affinities are similar across isoforms (Pumroy and Cingolani 2015; Smith et al. 2018; Marfori et al. 2012; Pumroy et al. 2015; Tsimbalyuk et al. 2022; Christie et al. 2016), it is proposed that specificity may instead be influenced by the linker region of bipartite NLSs (Fontes et al. 2000) or subtle sequence differences within non‐conserved regions of IMPα armadillo (ARM) repeats (Smith et al. 2018). Although human and chicken IMPα isoforms differ in amino acid sequence identity, ranging from 82% to 99% for *α*1 (82%), *α*3 (99%), *α*4 (98%), *α*5 (95%), *α*6 (94%), and *α*7 (94%) (Gabriel et al. 2011), our comparative analyses revealed that the residues forming the major and minor NLS binding sites are highly conserved (data not shown). Similarly, the sequence identity between chicken and mouse IMPβ1 is approximately 98% (data not shown), indicating functional conservation likely extends to nuclear import mechanisms across species.

One of the limitations of this study is that binding assays could not be performed with the full‐length UL36h protein due to its large size, the complexity of fluorescent labeling and associated technical challenges in recombinant expression. Instead, we used a fluorescently labeled synthetic peptide corresponding to the predicted NLS, which specifically interacted with nuclear import receptors, providing functional evidence for its role in nuclear import. Structural modeling indicates that this NLS is surface exposed in the full length protein, supporting the relevance of the peptide‐based assays. Future studies using truncated or full length constructs will be important to further define the mechanistic details of UL36h‐importin interactions.

In conclusion, our findings suggest that the predicted N‐terminal NLS of the GaAHV‐1 large tegument protein facilitates nuclear entry via an importin α/β1‐mediated pathway, with potential contributions from alternative nuclear transport mechanisms. These results underscore the likelihood of both species‐specific and multiple transport pathways are involved in herpesvirus nuclear localization. To fully understand this process, further research on subcellular localization experiments along with other potential nuclear localization mechanisms is recommended. Additionally, while this study focused on the GaAHV‐1 large tegument protein, it would be valuable to explore whether similar mechanisms exist in other gallid alphaherpesviruses by comparing the large tegument protein. Collectively, these findings advance our understanding of GaAHV‐1 biology, aiding in the development of antiviral therapies and rational vaccine design, and contributing to the virology research more broadly.

## Author Contributions

**Subir Sarker:** conceptualization, funding acquisition, methodology, project administration, resources, supervision, validation, writing – original draft, writing – review and editing. **Jade K. Forwood:** conceptualization, funding acquisition, methodology, project administration, resources, supervision, validation, writing – review and editing. **Babu Kanti Nath:** data curation, formal analysis, investigation, methodology, software, visualization, writing – original draft, writing – review and editing. **Crystall M. D. Swarbrick:** data curation, formal analysis, investigation, methodology, software, visualization, writing – original draft, writing – review and editing. **Daryl Ariawan:** methodology, resources, writing – review and editing. **Ole Tietz:** methodology, resources, writing – review and editing. **Reuben Blades:** methodology. **Gualtiero Alvisi:** methodology, writing – review and editing.

## Ethics Statement

The authors have nothing to report.

## Conflicts of Interest

None declared.

## Supporting information


**Supplementary Table S1:** Amino acids sequence similarities of UL36 gene from the selected herpesviruses. **Supplementary Table S2:** GaAHV‐1 NLS and NLS mutant peptides. **Supplementary Table S3:** Data collection and refinement statistics for structure of importin‐α2 in complex with GaAHV‐1 NLS. **Supplementary Table S4**: GaAHV‐1 NLS hydrogen bond and salt bridge interactions with IMPα2.

## Data Availability

The data that support the findings of this study are openly available in RCSB Protein Data Bank at https://www.rcsb.org/structure/unreleased/9MIK, reference number 9MIK.
