# Supplementary material for: Structural Insights Into the Nuclear Import of Gallid Alphaherpesvirus 1 Large Tegument Protein
Source: Microbiologyopen. 2026 Jan 22;15(1):e70216. doi: 10.1002/mbo3.70216 (PMC12826112; doi:10.1002/mbo3.70216)
Supplement: Supplementary file 1 — Supplementary Table S1: Amino acids sequence similarities of UL36 gene from the selected herpesviruses. Supplementary Table S2: GaAHV‐1 NLS and NLS mutant peptides. Supplementary Table S3: Data collection and refinement statistics for structure of importin‐α2 in complex with GaAHV‐1 NLS. Supplementary Table S4: GaAHV‐1 NLS hydrogen bond and salt bridge interactions with IMPα2. [file MBO3-15-e70216-s001.docx]

***Supplementary File***

**Structural insights into the nuclear import of gallid alphaherpesvirus 1 large tegument protein**

Babu Kanti Nath^1^, Crystall M. D. Swarbrick^1^, Reuben Blades^3^, Daryl Ariawan^3,^ Ole Tietz^3^, Gualtiero Alvisi^4^, Jade K. Forwood^1,2*^ and Subir Sarker^5,6,7*^

^1^Biosecurity Research Program and Training Centre, Gulbali Institute, Charles Sturt University, Wagga Wagga, NSW, Australia. [bnath@csu.edu.au](mailto:bnath@csu.edu.au); [cswarbrick@csu.edu.au](mailto:cswarbrick@csu.edu.au); [jforwood@csu.edu.au](mailto:jforwood@csu.edu.au)

^2^Training Hub Promoting Regional Industry and Innovation in Virology and Epidemiology, Gulbali Institute, Charles Sturt University, Wagga Wagga, New South Wales 2678, Australia. [jforwood@csu.edu.au](mailto:jforwood@csu.edu.au)

^3^Dementia Research Centre, Macquarie Medical School, Faculty of Medicine, Health and Human Sciences, Macquarie University, North Ryde, Sydney, NSW 2109, Australia. [reuben.blades@hdr.mq.edu.au](mailto:reuben.blades@hdr.mq.edu.au); [daryl.ariawan@mq.edu.au](mailto:daryl.ariawan@mq.edu.au); [ole.tietz@mq.edu.au](mailto:ole.tietz@mq.edu.au)

^4^Department of Molecular Medicine, University of Padua, Padua, Italy. [gualtiero.alvisi@unipd.it](mailto:gualtiero.alvisi@unipd.it)

^5^Biomedical Sciences & Molecular Biology, College of Medicine and Dentistry, James Cook University, Townsville, QLD 4811, Australia. [subir.sarker@jcu.edu.au](mailto:subir.sarker@jcu.edu.au)

^6^Australian Institute of Tropical Health and Medicine, James Cook University, Townsville, QLD 4811, Australia

^7^Department of Microbiology, Anatomy, Physiology and Pharmacology, School of Agriculture, Biomedicine and Environment, La Trobe University, Melbourne, Victoria 3086, Australia

* = Correspondence: [jforwood@csu.edu.au](mailto:jforwood@csu.edu.au); [subir.sarker@jcu.edu.au](mailto:subir.sarker@jcu.edu.au)

**Supplementary Table S1:** Amino acids sequence similarities of UL36 gene from the selected herpesviruses

|  |  | 1 | 2 | 3 | 4 | 5 | 6 | 7 | 8 | 9 | 10 | 11 | 12 | 13 | 14 | 15 | 16 | 17 | 18 |
| --- | --- | --- | --- | --- | --- | --- | --- | --- | --- | --- | --- | --- | --- | --- | --- | --- | --- | --- | --- |
| 1 | Gallid alphaherpesvirus 1 [HQ630064] [1] | 100 |  |  |  |  |  |  |  |  |  |  |  |  |  |  |  |  |  |
| 2 | Gallid alphaherpesvirus 2 [AF243438] [2] | 21.54 |  |  |  |  |  |  |  |  |  |  |  |  |  |  |  |  |  |
| 3 | Gallid alphaherpesvirus 3 [AB049735] [3] | 21.85 | 59.34 |  |  |  |  |  |  |  |  |  |  |  |  |  |  |  |  |
| 4 | Psittacid alphaherpesvirus 1 [NC_005264] [4] | 33.83 | 21.10 | 22.65 |  |  |  |  |  |  |  |  |  |  |  |  |  |  |  |
| 5 | Columbid alphaherpesvirus 1 [NC_034266] [5] | 22.85 | 42.42 | 43.13 | 22.92 |  |  |  |  |  |  |  |  |  |  |  |  |  |  |
| 6 | Human alphaherpesvirus 1 [JN555585] [6] | 22.08 | 29.03 | 30.41 | 22.19 | 33.46 |  |  |  |  |  |  |  |  |  |  |  |  |  |
| 7 | Human alphaherpesvirus 2 [JN561323] [7] | 21.90 | 28.80 | 30.73 | 22.45 | 33.14 | 82.35 |  |  |  |  |  |  |  |  |  |  |  |  |
| 8 | Cacatuid alphaherpesvirus 2 [MK360902] [8] | 34.37 | 21.45 | 21.82 | 48.08 | 21.07 | 20.07 | 20.64 |  |  |  |  |  |  |  |  |  |  |  |
| 9 | Macropodid alphaherpesvirus 4 [MT900474] [9] | 21.35 | 26.89 | 28.20 | 20.37 | 30.21 | 50.13 | 49.39 | 20.50 |  |  |  |  |  |  |  |  |  |  |
| 10 | Macropodid alphaherpesvirus 2 [MT900475] [10] | 21.17 | 26.71 | 28.46 | 20.41 | 29.93 | 50.10 | 49.31 | 20.28 | 94.82 |  |  |  |  |  |  |  |  |  |
| 11 | Macropodid alphaherpesvirus 1 [NC_029132] [11] | 20.57 | 26.74 | 27.97 | 20.43 | 30 | 49.56 | 49.47 | 20.37 | 81.54 | 81.14 |  |  |  |  |  |  |  |  |
| 12 | Elephantid betaherpesvirus 3A [MN373268] [12] | 16.74 | 15.06 | 16.91 | 19.18 | 17.80 | 17.92 | 17.93 | 18.82 | 17.11 | 17.61 | 17.85 |  |  |  |  |  |  |  |
| 13 | Elephantid betaherpesvirus 4 [NC_028379] [13] | 16.61 | 15.20 | 16.81 | 18.58 | 17.31 | 17.85 | 18.02 | 18.29 | 16.94 | 17.33 | 17.58 | 87.73 |  |  |  |  |  |  |
| 14 | Elephantid betaherpesvirus 2 [MZ822421] [14] | 14.40 | 15.85 | 16.35 | 16.48 | 15.95 | 16.58 | 16.70 | 16.50 | 16.12 | 16.12 | 16.25 | 47.73 | 46.90 |  |  |  |  |  |
| 15 | Elephantid betaherpesvirus 5 [NC_024696] [15] | 14.85 | 15.65 | 15.99 | 16.16 | 16.08 | 16.82 | 16.84 | 16.85 | 15.38 | 15.44 | 15.30 | 48.82 | 47.72 | 79.76 |  |  |  |  |
| 16 | Vombatid gammaherpesvirus 1 [NC_055554] [16] | 17.89 | 17.90 | 17.88 | 17.54 | 18.47 | 14.62 | 15.16 | 18.43 | 15.41 | 15.85 | 15.35 | 17.18 | 17.33 | 16.80 | 17.73 |  |  |  |
| 17 | Elephantid betaherpesvirus 1 [KC462165] [17] | 15.36 | 14.99 | 15.47 | 16.28 | 16.28 | 15.64 | 15.87 | 16.28 | 15.40 | 15.62 | 15.75 | 46.39 | 45.79 | 62.27 | 63.86 | 17.68 |  |  |
| 18 | Abalone herpesvirus [JX453331] [18] | 16.90 | 16.38 | 16.19 | 17.06 | 16.59 | 15.50 | 14.49 | 16.21 | 15.44 | 15.30 | 15.47 | 15.22 | 15.18 | 13.59 | 13.91 | 21.24 | 14.12 |  |

**Supplementary Table S2: GaAHV-1 NLS and NLS mutant peptides**

| **Peptides** | **Sequences** |
| --- | --- |
| NLS | ^296^DRRKAIAPWSVPVRPRSKKRQKPQ^319^ |
| **Mutant peptides** | **Sequences** |
| R298A | ^296^DRAaAIAPWSVPVRPRSKKRQKPQ^319^ |
| Δ296-308 | ^296^------------------------RPRSKKRQKPQ^319^ |
| Δ296-308; K314A | ^296^------------------------RPRSKaRQKPQ^319^ |
| Δ296-308; R315A | ^296^------------------------RPRSKKaQKPQ^319^ |
| Δ296-308; K317A | ^296^------------------------RPRSKKRQaPQ^319^ |

**Supplementary Table S3:** Data collection and refinement statistics for structure of importin-α2 in complex with GaAHV-1 NLS

| **GaAHV-1 NLS and importin-α2 (PDB Code: 9MIK)** | |
| --- | --- |
| Data Collection (high resolution statistics in parentheses) | |
| Wavelength | 0.95374 |
| Data-collection temperature (K) | 298 |
| Detector Type | Dectris EIGER X 16M |
| Detector | Pixel |
| Resolution range (Å) | 44.96 – 2.60 |
| Space group | P2_1_ 2_1_ 2_1_ |
| Unit cell (Å) ; (^o^) | 78.09 89.92 99.20; 90 90 90 |
| Total reflections | 125753 (15709) |
| Unique reflections | 21815 (2627) |
| Multiplicity | 5.8 (6.0) |
| Completeness (%) | 99.0 (99.6) |
| Mean I/σ (I) | 13.5 (4.0) |
| Wilson B-factor Å2 | 39.00 |
| R_pim_ | 0.038 (0.216) |
| **Refinement** |  |
| R_work_ | 0.1754 |
| R_free_ | 0.2123 |
| No. of non-hydrogen atoms | 6766 |
| Macromolecules | 2 |
| Solvent | 30 |
| Protein residues | 434 |
| Bond length r.m.s.d (Å) | 0.003 |
| Bond angle r.m.s.d (^o^) | 0.554 |
| Ramachandran favoured (%) | 97.90 |
| Ramachandran allowed (%) | 2.10 |
| Ramachandran outliers (%) | 0.00 |

**Supplementary Table S4**: GaAHV-1 NLS hydrogen bond and salt bridge interactions with IMPα2

| **Hydrogen bonds** | |
| --- | --- |
| **IMPα2** | **GaAHV-1 NLS2** |
| ASN 361[HD21] | ARG 298[O] |
| ASN 235[HD21] | LYS 313[O] |
| ASN 188[HD21] | ARG 315[O] |
| ASN 146[HD21] | LYS 317[O] |
| VAL 321[O] | ARG 297[HH11] |
| ASP 325[OD1] | ARG 297[HH21] |
| THR 328[OG1] | ARG 297[HH22] |
| ASN 361[OD1] | ARG 298[H] |
| GLU 396[OE1] | ARG 298[HH12] |
| GLU 396[OE2] | ARG 298[HH22] |
| GLY 281[O] | LYS 299[HZ2] |
| THR 322[OG1] | LYS 299[HZ3] |
| GLY 150[O] | LYS 314[HZ1] |
| THR 155[OG1] | LYS 314[HZ2] |
| ASP 192[OD1] | LYS 314[HZ2] |
| ASN 188[OD1] | ARG 315[H] |
| ASN 228[OD1] | ARG 315[HH12] |
| ASN 146[OD1] | LYS 317[H] |
| **Salt Bridges** | |
| **IMPα2** | **GaAHV-1 NLS2** |
| ASP 325[OD1] | ARG 297[NH2] |
| GLU 396[OE2] | ARG 298[NH1] |
| GLU 396[OE1] | ARG 298[NH1] |
| GLU 396[OE2] | ARG 298[NH2] |
| GLU 396[OE1] | ARG 298[NH2] |
| ASP 192[OD1] | LYS 314[NZ] |

*
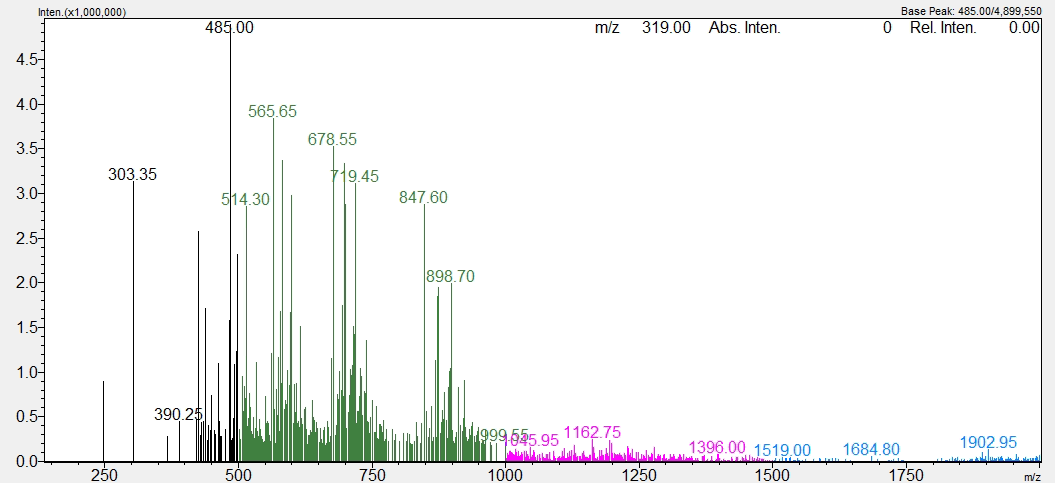
*

**Supplementary Figure S1**: Peptide synthesis of NLS. Molecular Weight: 3387.048 g mol-1; LCMS [ESI+]: 847.60 (m/z4; calculated: 847.77), 678.55 (m/z5; calculated: 678.42), 565.65 (m/z6; calculated: 565.52), 485.00 (m/z7; calculated: 484.87); Purity >95%.


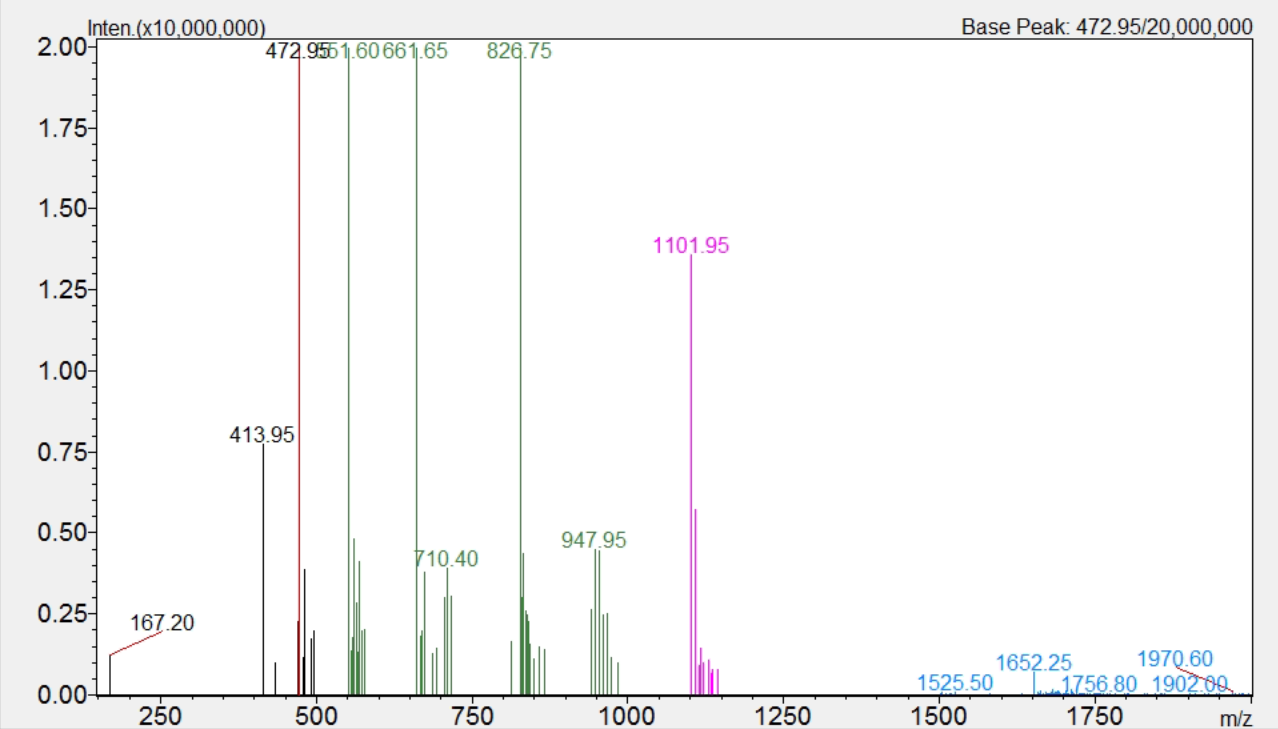


**Supplementary Figure 2**: Peptide synthesis of mutant R298A. Molecular Weight: 3301.94 g mol-1. LCMS [ESI+]: 1652.25 (m/z2; calculated: 1651.98), 1101.95 (m/z3; calculated: 1101.65), 826.75 (m/z4; calculated: 826.49), 661.65 (m/z5; calculated: 661.40), 551.60 (m/z6; calculated: 551.33), 472.95 (m/z7; calculated: 472.71), 413.95 (m/z8; calculated: 413.75). Purity >95%.


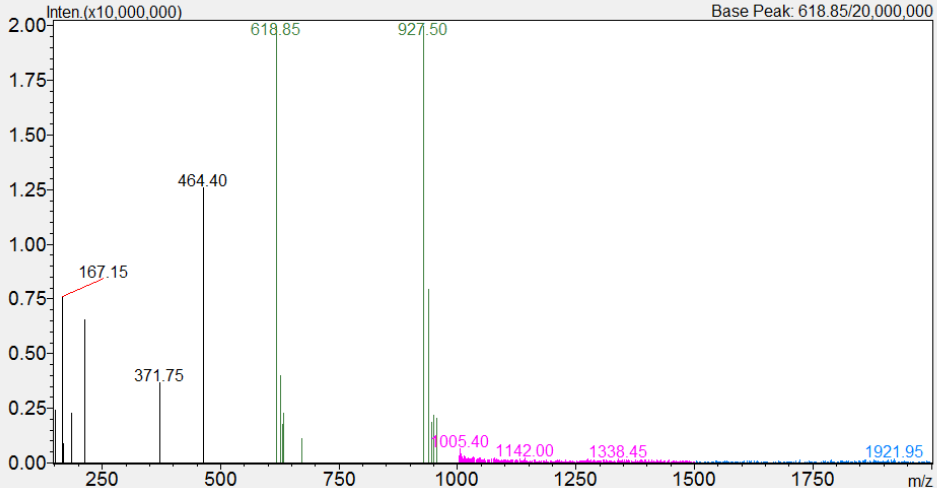


**Supplementary Figure 3**: Peptide synthesis of mutant K314A; Molecular Weight: 1853.201 g mol-1; LCMS [ESI+]: 927.50 (m/z2; calculated: 927.61), 618.85 (m/z3; calculated: 618.74), 464.40 (m/z4; calculated: 464.31); Purity >95%.


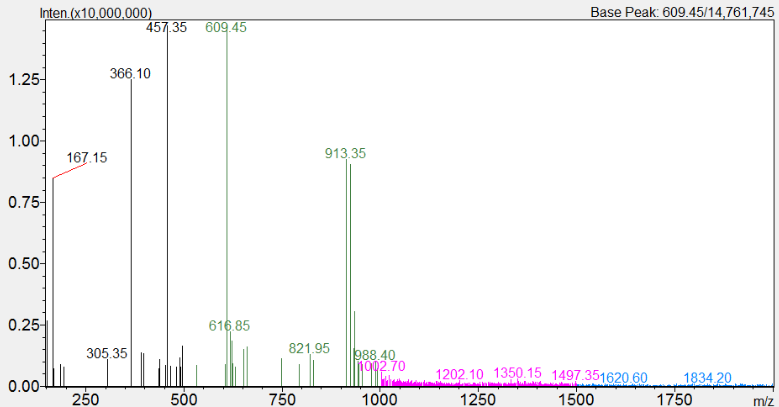


**Supplementary Figure 4**: Peptide synthesis of mutant R315A; Molecular Weight: 1825.187 g mol-1; LCMS [ESI+]: 913.35 (m/z2; calculated: 913.60), 609.45 (m/z3; calculated: 609.40), 457.35 (m/z4; calculated: 457.30), 366.10 (m/z5; calculated: 366.05); Purity >95%.


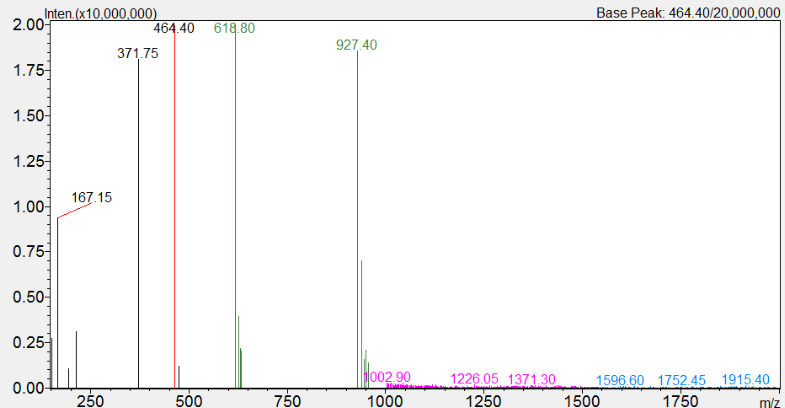


**Supplementary Figure 5**: Peptide synthesis of mutant K317A. Molecular Weight: 1853.201 g mol-1; LCMS [ESI+]: 927.40 (m/z2; calculated: 927.61), 618.80 (m/z3; calculated: 618.74), 464.40 (m/z4; calculated: 464.31), 371.75 (m/z5; calculated: 371.65); Purity >95%.


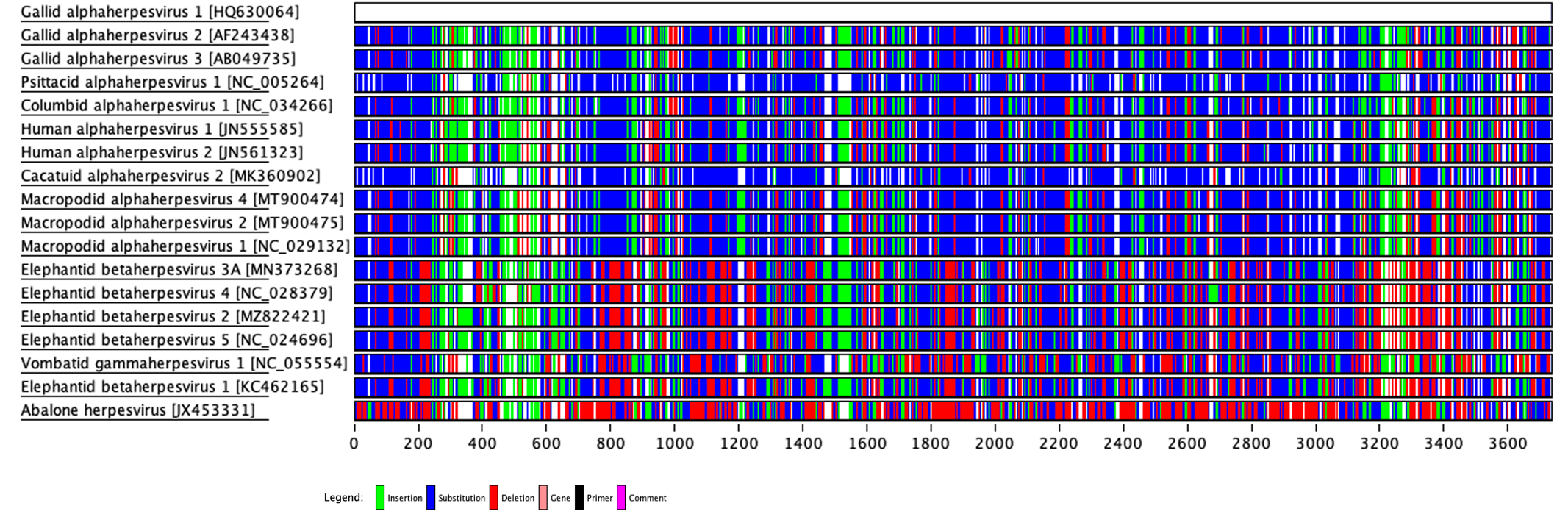


**Supplementary Figure 6**: A comparative visualization of **UL36** gene amino acid sequences from selected herpesviruses was carried out using Base-By-Base. Sequence variations within the **UL36** gene of GaAHV-1 are color-coded: green indicates insertions, blue denotes substitutions, red represents deletions, black identifies primers, and pink highlights user comments.

**
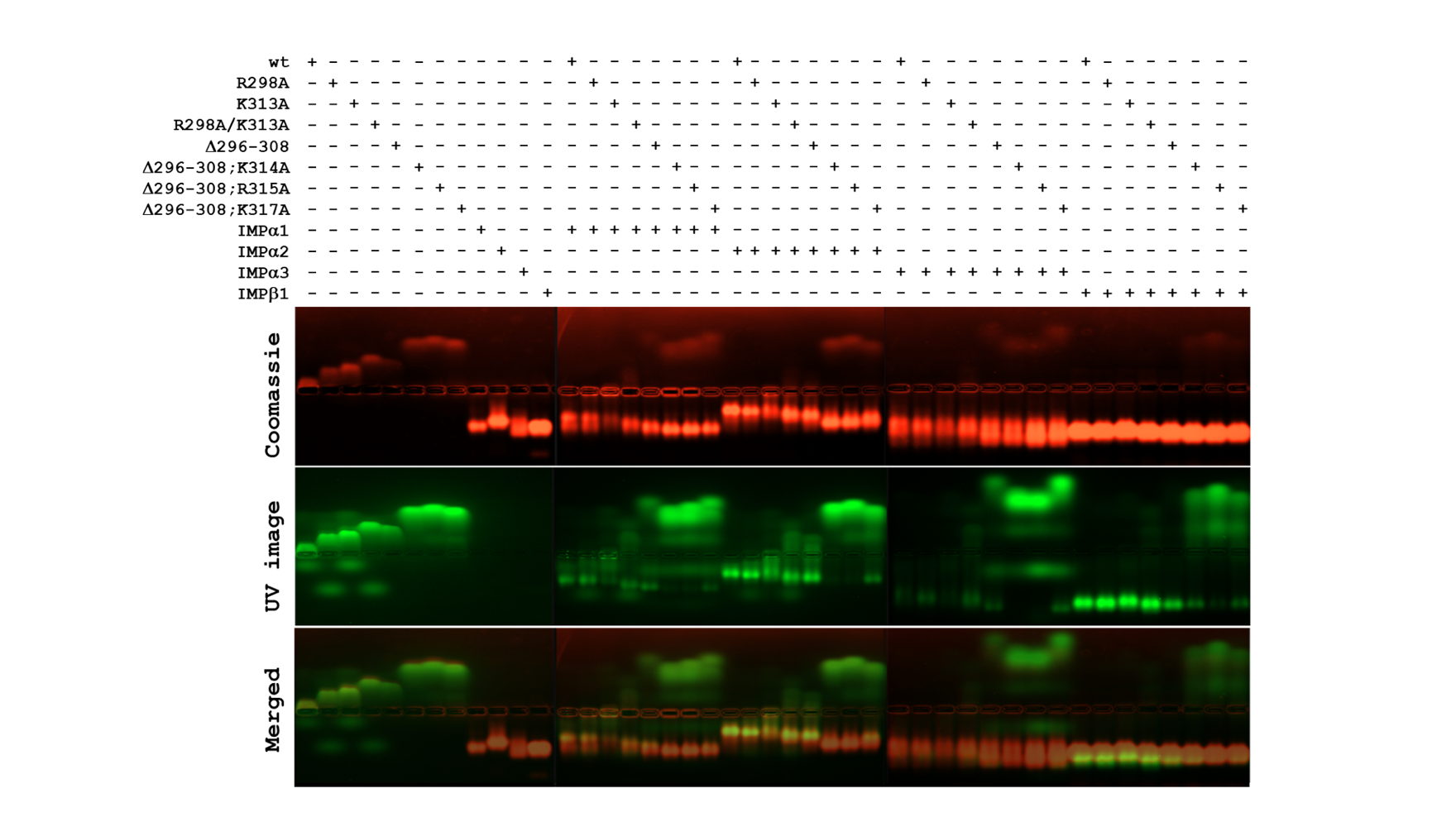
**

**Supplementary Figure 7:** An extended image of figure 4B. EMSA showing poor binding affinity of GaAHV-1 NLS mutant peptides to IMPα isoforms and IMPβ1. Protein bands were visualized post-electrophoresis using Coomassie Blue staining (red) (top panel). The peptides contain a FITC and Ahx linker and were visualized by excitation with an UV lamp (green) (middle panel). Overlay image of UV and Coomassie stained image is shown in bottom panel. The EMSA results reflect outcomes from three independent replicates.
